# Supplementary material for: Wnt-Ror-Dvl signalling and the dystrophin complex organize planar-polarized membrane compartments in C. elegans muscles
Source: Nat Commun. 2024 Jun 10;15:4935. doi: 10.1038/s41467-024-49154-8 (PMC11164867; doi:10.1038/s41467-024-49154-8)
Supplement: Supplementary file 1 — Supplementary Information [file 41467_2024_49154_MOESM1_ESM.pdf]

nature communications

Wnt-Ror-Dvl signalling and the dystrophin complex organize planar-polarized membrane compartments in *C. elegans* muscles

Alice Peysson*, Noura Zariohi*, Marie Gendrel, Amandine Chambert-Loir, Noémie Frébault, Elise Cheynet, Olga Andrini, Thomas Boulin

Supplementary Information

Supplementary Figures S1, S2, S3, S4, S5, S6, S7 and Supplementary Table 1

Figure S1

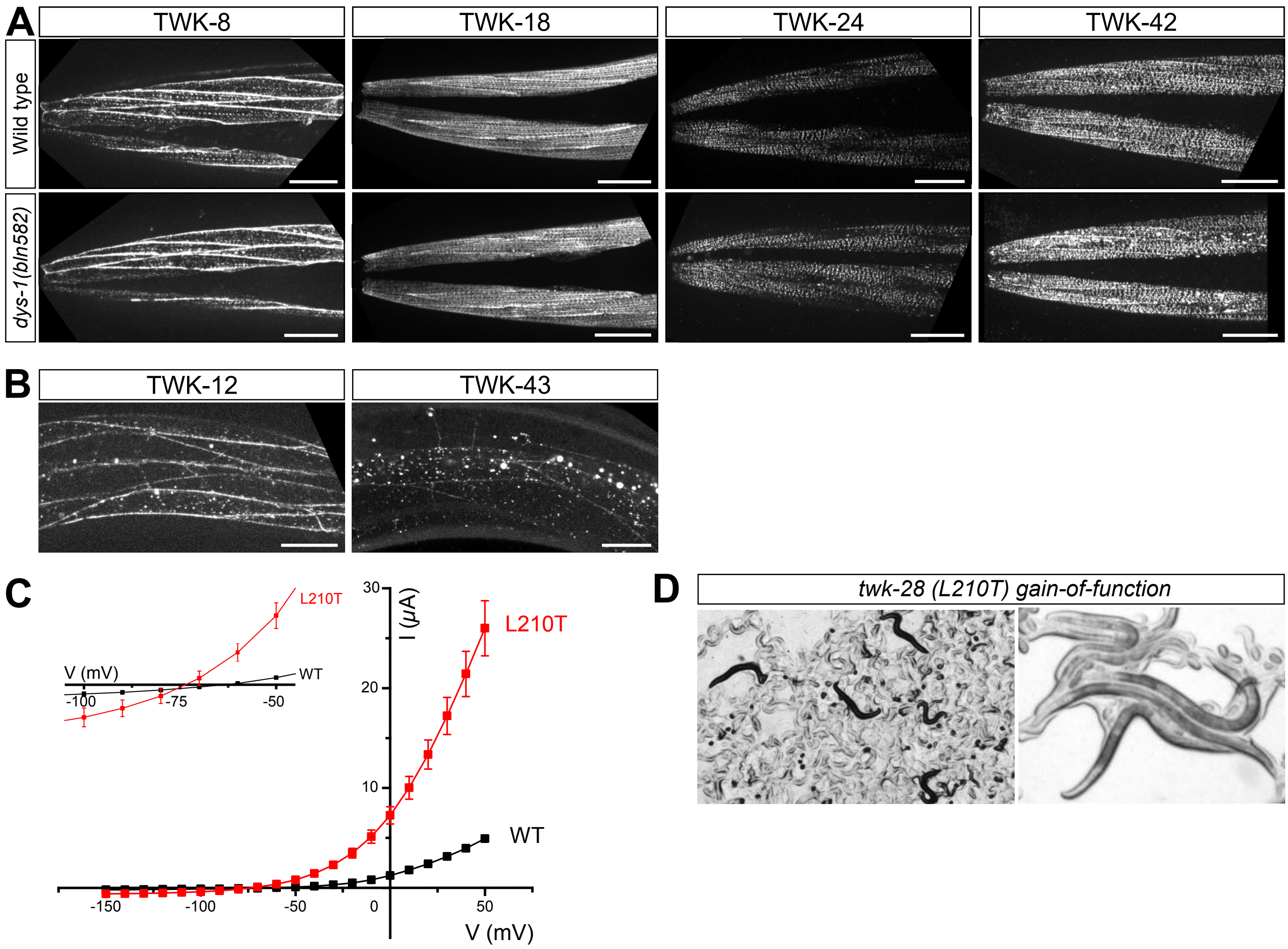

E

Allele	Locus	Mutation	Effect
bln557	*dys-1*	G to A ; ChrI:11556770	Q30Ochre
bln545	*dys-1*	C to T ; ChrI:11556616	W81Amber
bln582	*dys-1*	C to T ; ChrI:11556528	W110Opal
bln554	*dys-1*	C to T ; ChrI:11556280	W138Amber
bln583	*dys-1*	T to A ; ChrI:11544157	K1527Amber
bln568	*dys-1*	G to A ; ChrI:11543893	Q1615Ochre
bln552	*dys-1*	C to T ; ChrI:11540064	W2277Opal
bln571	*dys-1*	C to A ; ChrI:11538895	E2442Ochre
bln565	*dys-1*	C to T ; ChrI:11538087	W2582Opal
bln573	*dys-1*	G to A ; ChrI:11537052	Q2695Ochre
bln566	*dys-1*	C to T ; ChrI:11536816	W2773Opal
bln564	*dys-1*	C to T ; ChrI:11536387	W2807Amber
bln562	*dys-1*	G to A ; ChrI:11534654	Q2992Ochre
bln556	*dys-1*	G to A ; ChrI:11532291	loss of splice-donor
bln549	*islo-1*	G to A ; ChrIV:8137939	G165D

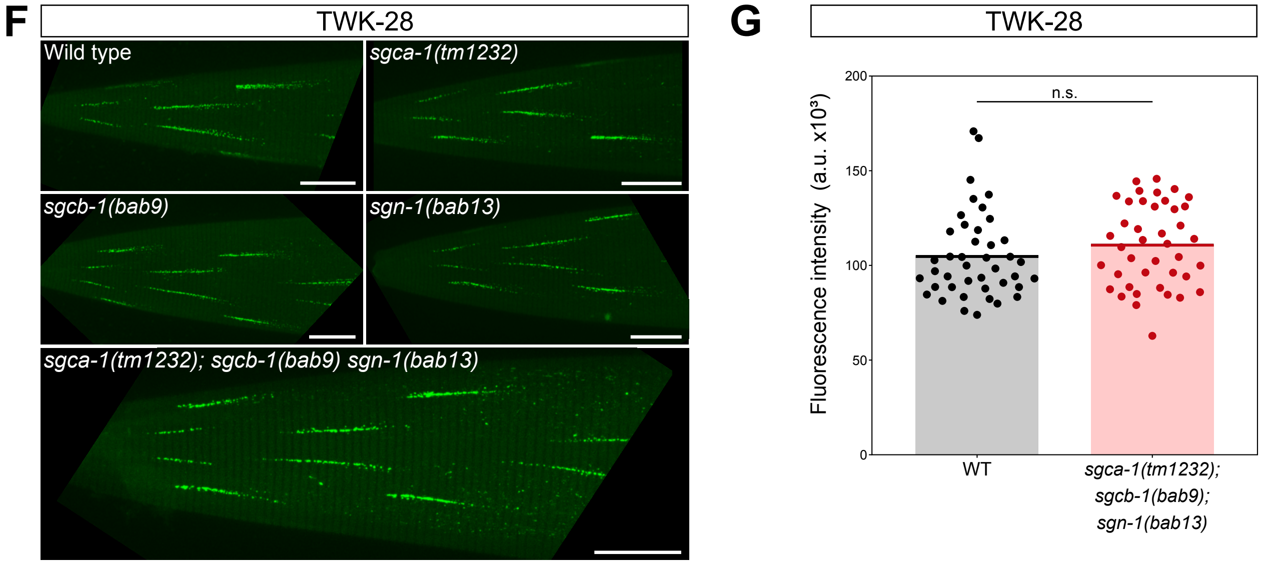

Figure S1. Genetic suppression of a TWK-28 L210T gain-of-function mutant by mutations in *dys-1* and *islo-1*

(A) Two-pore domain potassium channels TWK-8, TWK-18, TWK-24, and TWK-42 expression and subcellular distributions are unchanged in *dys-1(bln582)* loss-of-function mutants. Representative images of head musculature.

(B) Two-pore domain potassium channels TWK-12 and TWK-43 are enriched in muscle arms and on the lateral membrane of muscle cells.

(C) Current–voltage relationships obtained at pH 7.4 in *X. laevis* oocytes after injection of cRNA encoding TWK-28 wild-type (WT, black squares) and TWK-28 L210T gain-of-function channels (L210T, red squares). Inset shows leftward shift of reversal potential in TWK-28 L210T. n = 8 and n = 7 for wild type and TWK-28 L210T, respectively.

(D) Low and high magnification micrographs illustrating reduced locomotion and relaxed body posture of TWK-28 L210T gain-of-function mutants on NGM plates.

(E) Loss-of-function mutations in *dys-1* and a missense mutation in *islo-1* suppress paralysis of the TWK-28 L210T gain-of-function mutation.

(F) TWK-28 localization and levels are unchanged in sarcoglycan single or triple null mutants.

(G) Quantification of TWK-28-associated fluorescence. n = 42, 41 (for respective columns), N = 3 independent experiments. Mann-Whitney test; p = 0.1299, n.s., not significant.

Source data and statistical analysis are presented in the Source Data file. Scale bars, 20 µm

Figure S2

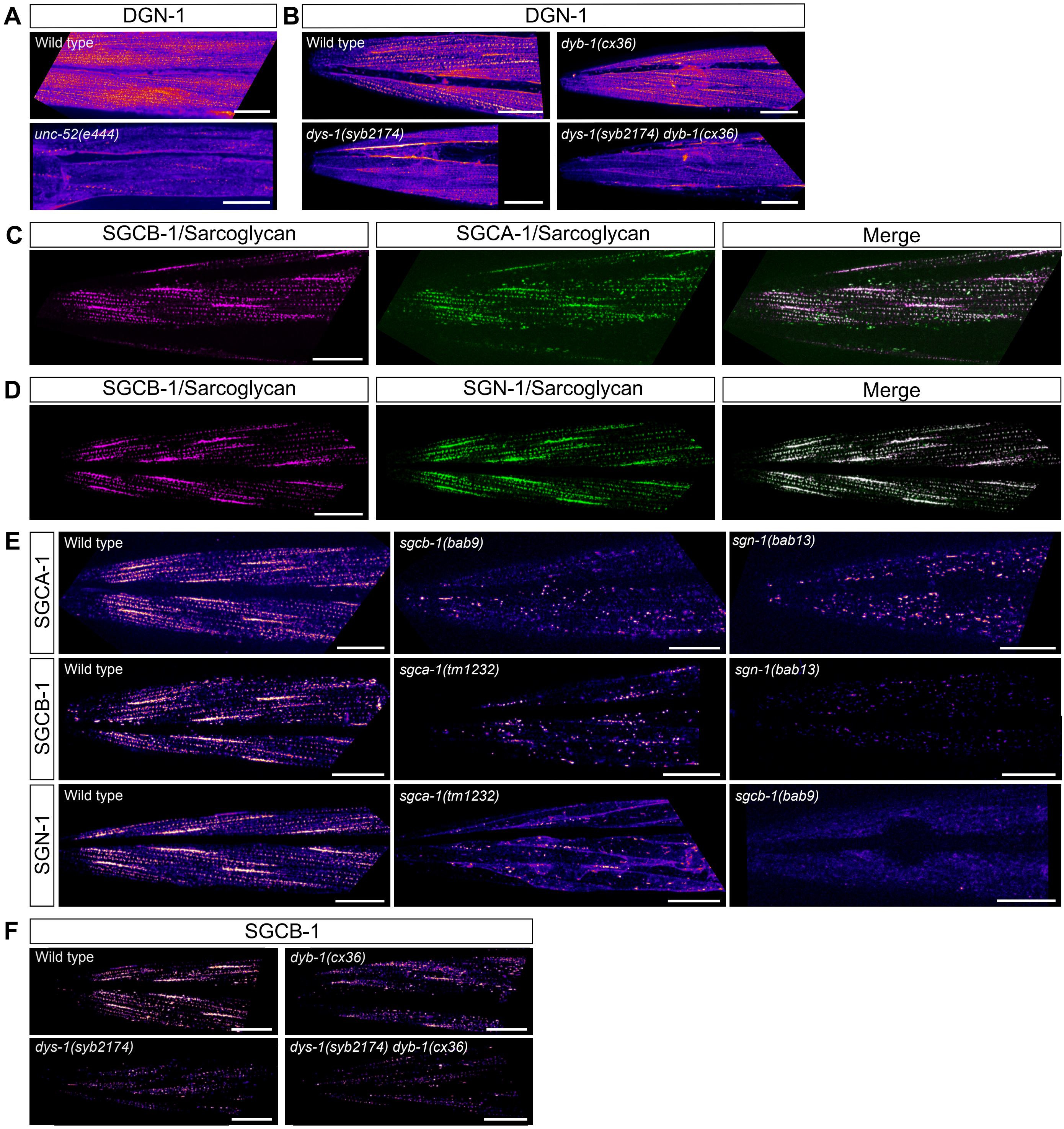

A
DGN-1
Wild type
unc-52(e444)
B
DGN-1
Wild type
dyb-1(cx36)
dys-1(syb2174)
dys-1(syb2174) dyb-1(cx36)
C
SGCB-1/Sarcoglycan
SGCA-1/Sarcoglycan
Merge
D
SGCB-1/Sarcoglycan
SGN-1/Sarcoglycan
Merge
E
SGCA-1
Wild type
sgcb-1(bab9)
sgn-1(bab13)
SGCB-1
Wild type
sgca-1(tm1232)
sgn-1(bab13)
SGN-1
Wild type
sgca-1(tm1232)
sgcb-1(bab9)
F
SGCB-1
Wild type
dyb-1(cx36)
dys-1(syb2174)
dys-1(syb2174) dyb-1(cx36)

Figure S2. Dystrophin is required for sarcolemmal localization of dystroglycan and sarcoglycans.

(A) Reduction in surface expression and mislocalization of DGN-1/Dystroglycan in *unc-52/*perlecan loss-of-function mutants.

(B) DGN-1/Dystroglycan localization is dependent on DYS-1, but not DYB-1.

(C), (D) Sarcoglycans SGCA-1 and SGN-1 are colocalized with SGCB-1.

(E) Subunit interdependence for surface expression of SGCA-1, SGCB-1 and SGN-1.

(F) SGBC-1 localization is disrupted differently in *dys-1* and *dyb-1* mutants.

Scale bars, 20 μm.

Figure S3

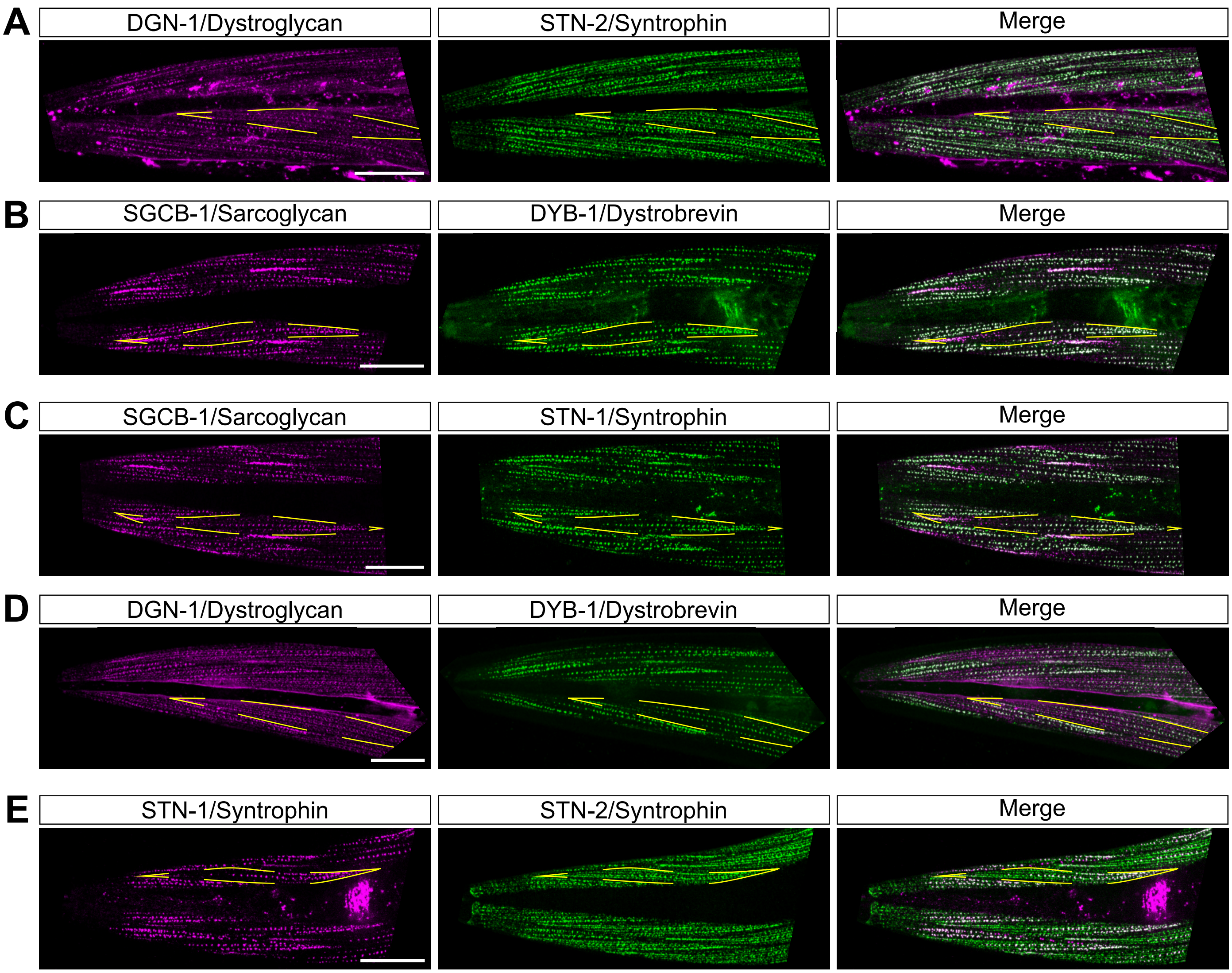
A
DGN-1/Dystroglycan
STN-2/Syntrophin
Merge
B
SGCB-1/Sarcoglycan
DYB-1/Dystrobrevin
Merge
C
SGCB-1/Sarcoglycan
STN-1/Syntrophin
Merge
D
DGN-1/Dystroglycan
DYB-1/Dystrobrevin
Merge
E
STN-1/Syntrophin
STN-2/Syntrophin
Merge

Figure S3. Relative localization of DAPC component in *C. elegans* head muscles
(A) Colocalization of DGN-1 and STN-2.
(B) Colocalization of SGCB-1 and DYB-1.
(C) Colocalization of SGCB-1 and STN-1.
(D) Colocalization of DGN-1 and DYB-1.
(E) Colocalization of STN-1 and STN-2.
Muscle cell outlines are indicated with yellow dashed lines. Scale bars, 20 μm.

Figure S4

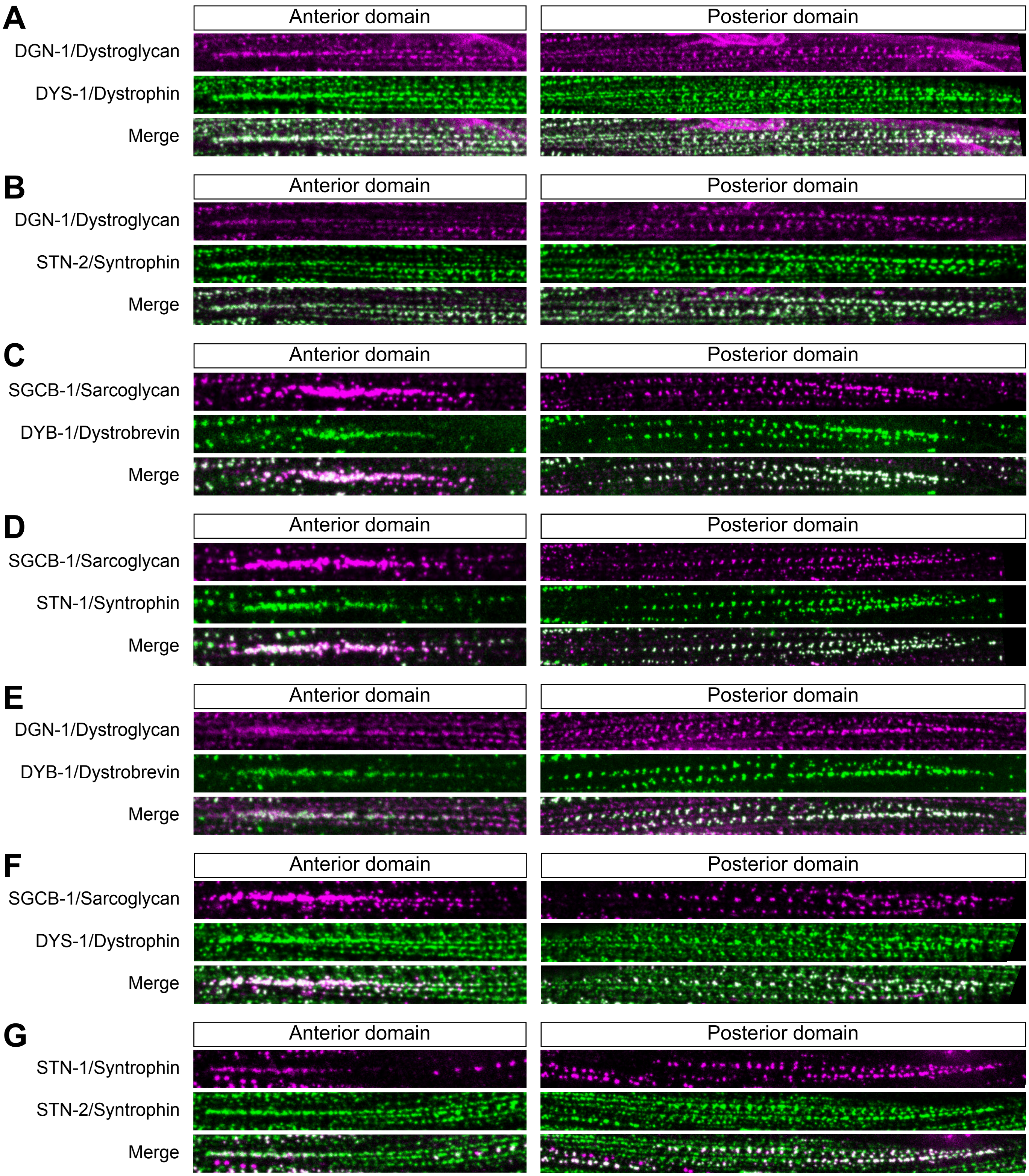
A
Anterior domain
Posterior domain
DGN-1/Dystroglycan
DYS-1/Dystrophin
Merge
B
Anterior domain
Posterior domain
DGN-1/Dystroglycan
STN-2/Syntrophin
Merge
C
Anterior domain
Posterior domain
SGCB-1/Sarcoglycan
DYB-1/Dystrobrevin
Merge
D
Anterior domain
Posterior domain
SGCB-1/Sarcoglycan
STN-1/Syntrophin
Merge
E
Anterior domain
Posterior domain
DGN-1/Dystroglycan
DYB-1/Dystrobrevin
Merge
F
Anterior domain
Posterior domain
SGCB-1/Sarcoglycan
DYS-1/Dystrophin
Merge
G
Anterior domain
Posterior domain
STN-1/Syntrophin
STN-2/Syntrophin
Merge

Figure S4. DAPC components partition to dystroglycan- or sarcoglycan-containing membrane compartments

(A) Colocalization of DGN-1 and DYS-1.
(B) Colocalization of DGN-1 and STN-2.
(C) Colocalization of SGCB-1 and DYB-1.
(D) Colocalization of SGCB-1 and STN-1.
(E) Colocalization of DGN-1 and DYB-1.
(F) Colocalization of SGCB-1 and DYS-1.
(G) Colocalization of STN-1 and STN-2.
Anterior domain, comet-shaped domain at anterior tip of muscle cells; *Posterior domain,* punctate clustered pattern in posterior part of muscle cells.

Figure S5

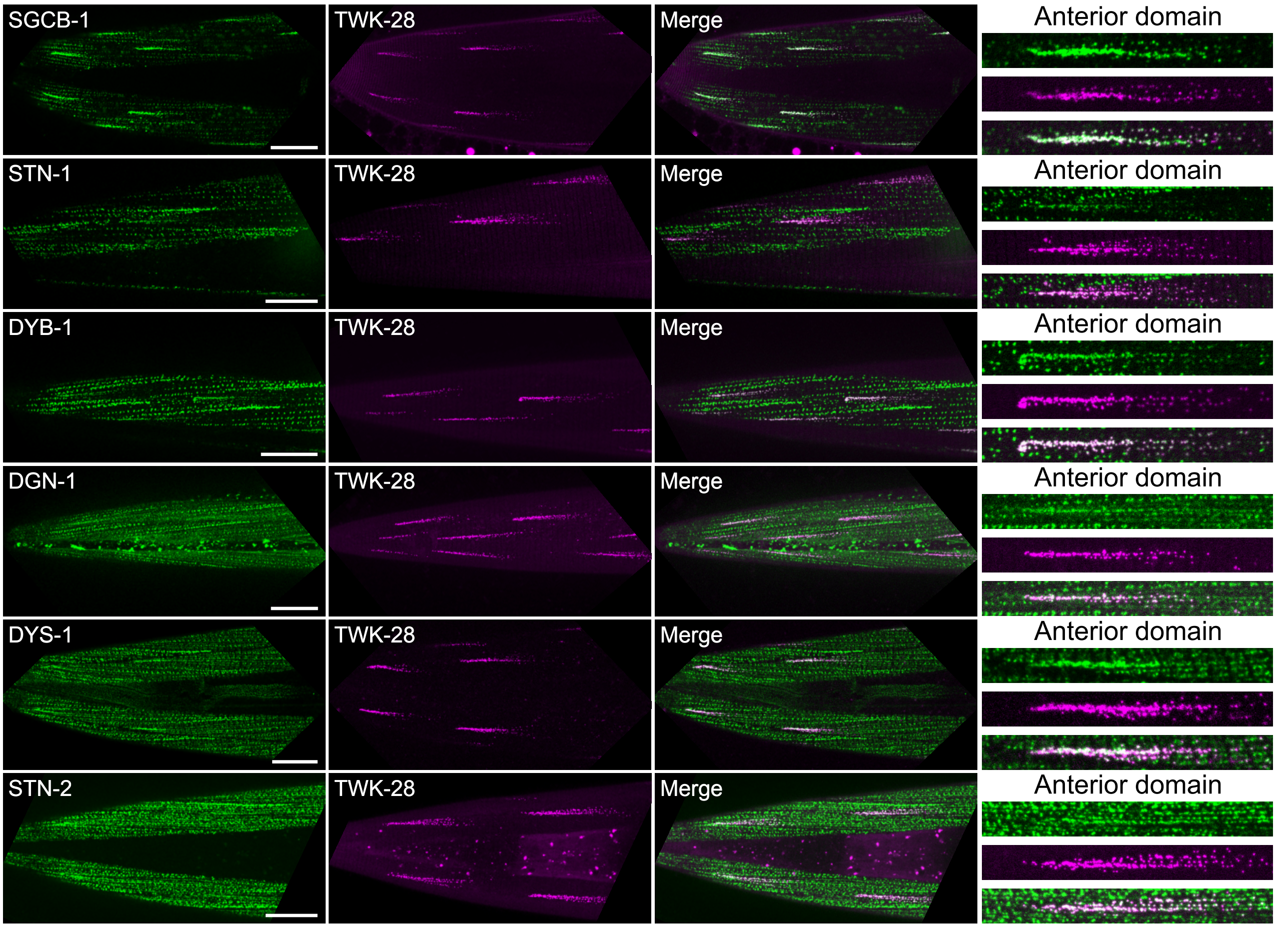
SGCB-1
TWK-28
Merge
Anterior domain
STN-1
TWK-28
Merge
Anterior domain
DYB-1
TWK-28
Merge
Anterior domain
DGN-1
TWK-28
Merge
Anterior domain
DYS-1
TWK-28
Merge
Anterior domain
STN-2
TWK-28
Merge
Anterior domain

Figure S5. TWK-28 colocalizes with all DAPC components
Colocalization of TWK-28 with SGCB-1, STN-1, DYB-1, DGN-1, DYS-1 and STN-2. Rightmost column, magnified view of anterior region of individual muscle cells.
Scale bars, 20 μm.

Figure S6

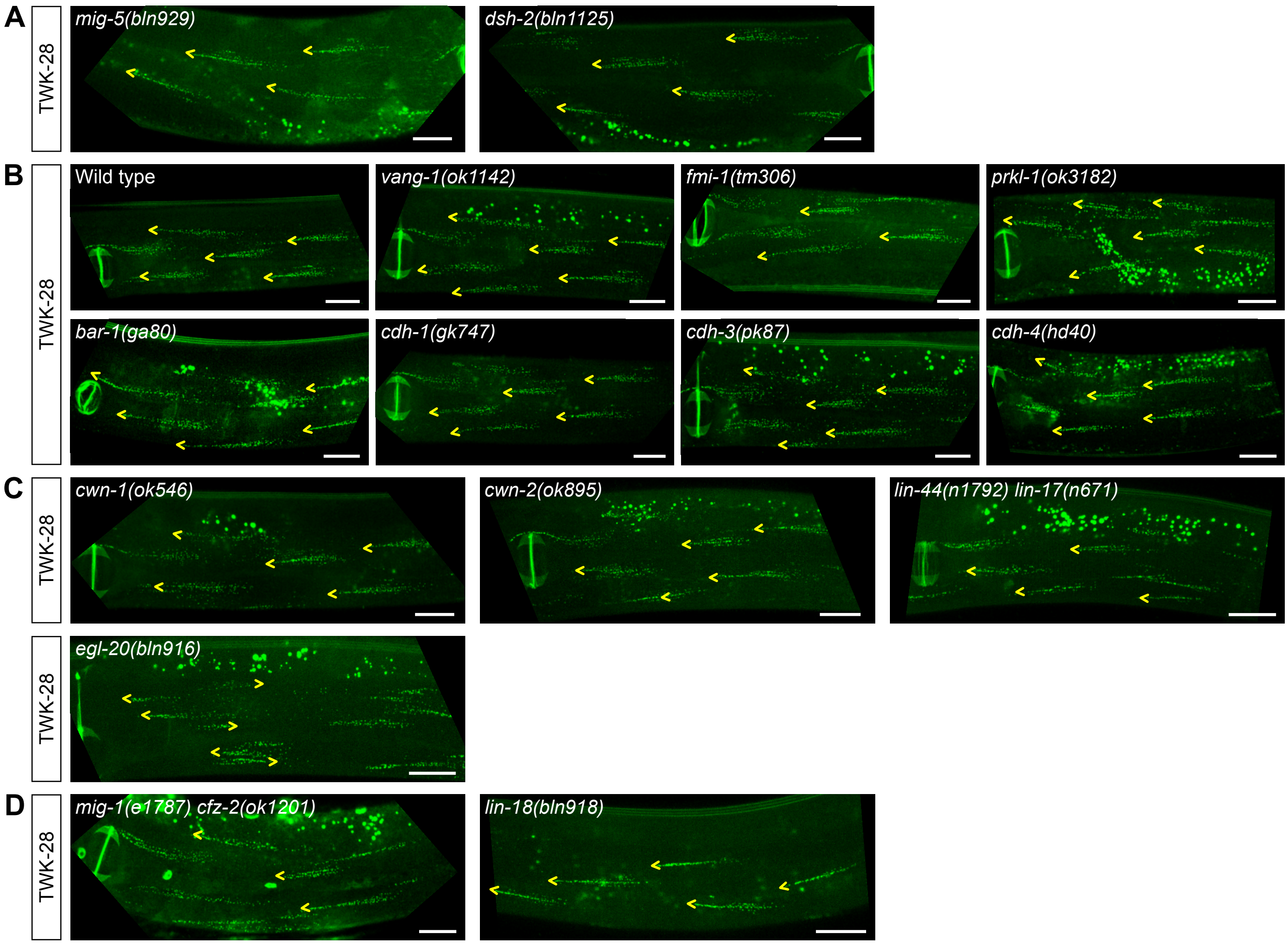
A
TWK-28
mig-5(bln929)
dsh-2(bln1125)
B
TWK-28
Wild type
vang-1(ok1142)
fmi-1(tm306)
prkl-1(ok3182)
bar-1(ga80)
cdh-1(gk747)
cdh-3(pk87)
cdh-4(hd40)
C
TWK-28
cwn-1(ok546)
cwn-2(ok895)
lin-44(n1792) lin-17(n671)
TWK-28
egl-20(bln916)
D
TWK-28
mig-1(e1787) cfz-2(ok1201)
lin-18(bln918)

Figure S6. EGL-20/Wnt is required for muscle cell polarity while several Wnt ligands and receptors are not

(A) Polarized localisation of TWK-28 is unaffected in *mig-5* or *dsh-2* null mutants. Homozygous *dsh-2* mutants were derived from heterozygous parents.

(B) Polarized localisation of TWK-28 is maintained in the absence of core planar cell polarity components (*vang-1, fmi-1, prkl-1*), Cadherin/Fat/Dachsous pathway components (*cdh-1, cdh-3, cdh-4*), or β-catenin (*bar-1*).

(C), (D) Wnt ligands (*cwn-1, cwn-2, lin-44*), frizzled receptors (*lin-17, mig-1, cfz-2*) and the tyrosine kinase-related receptors LIN-18/Ryk are not required for TWK-28 polarization. *egl-20(bln916)* –an early nonsense mutation at position Pro21– disrupts TWK-28 polarity in posterior midbody muscles.

Scale bars, 20 μm.

Figure S7

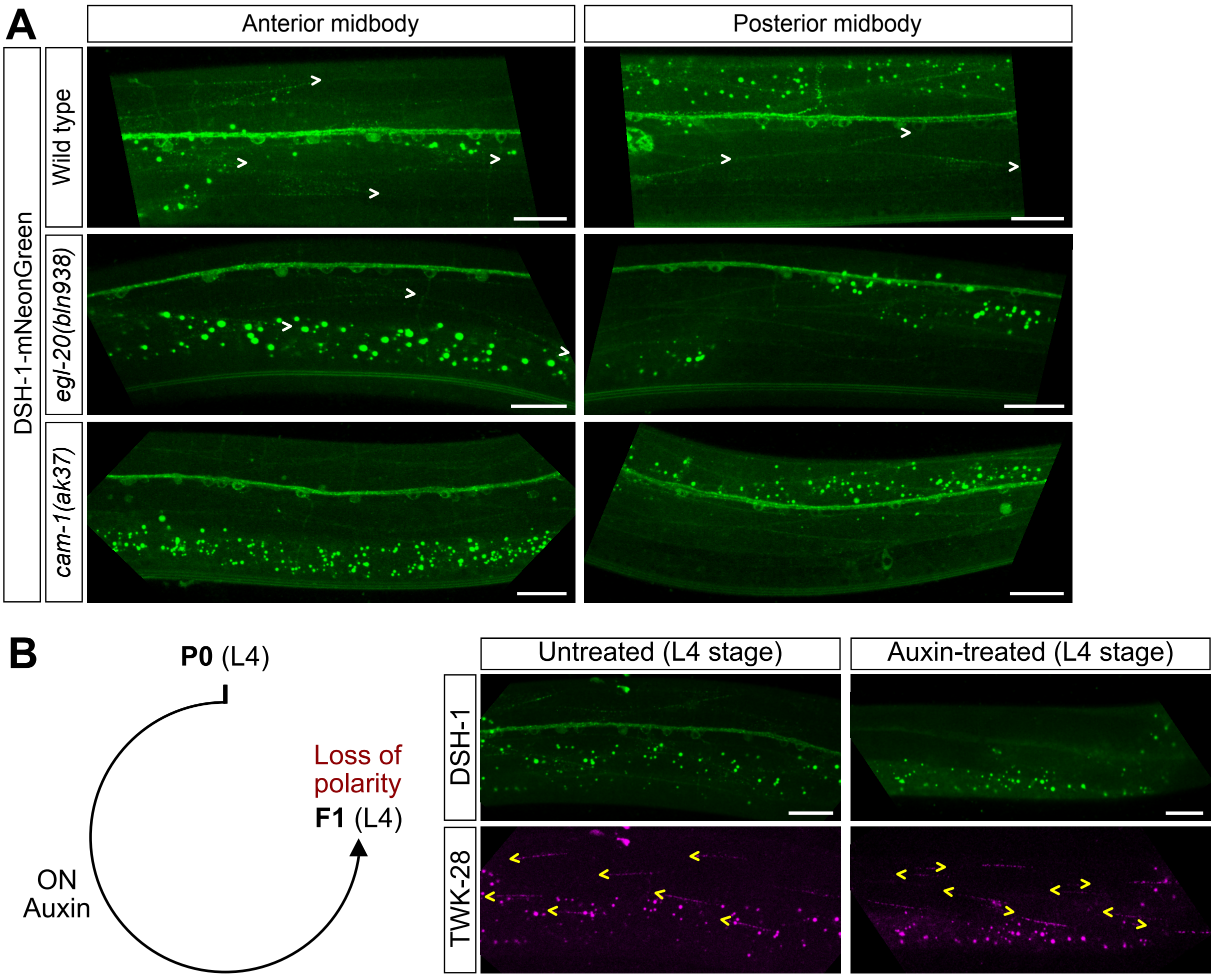
A
Anterior midbody
Posterior midbody
DSH-1-mNeonGreen
Wild type
egl-20(bln938)
cam-1(ak37)
B
P0 (L4)
Loss of polarity
F1 (L4)
ON Auxin
Untreated (L4 stage)
Auxin-treated (L4 stage)
DSH-1
TWK-28

Figure S7. The localization of DSH-1 is dependent on CAM-1 and EGL-20

(A) Enrichment of DSH-1 at the posterior end of muscle cells is lost in *cam-1* and *egl-20* mutants. DSH-1 localization is however conserved in the anterior midbody of *egl-20* mutants, consistent with the posteriorly restricted distribution of EGL-20. White arrowheads indicate DSH-1 enrichment at the posterior end of muscle cells. Ventral views.

(B) Life-long degradation (Auxin-treated) of DSH-1-AID-mNeonGreen using a ubiquitous TIR1-expressing transgene disrupts asymmetric localization of TWK-28 in muscle cells. Yellow arrowheads indicate the position and orientation of TWK-28 domains at the extremities of muscle cells.

Scale bars, 20 µm.

Supplementary Table 1: Conservation of DAPC components in vertebrates and *C. elegans*

Vertebrates	***C. elegans***
Dystrophin, Utrophin, DRP2	DYS-1
Dystroglycan (DAG1)	DGN-1
Dystrobrevin α,β	DYB-1
Syntrophin-α1,-β1,-β2	STN-1
Syntrophin -γ1,-γ2	STN-2
Sarcoglycan-α,-ε	SGCA-1
Sarcoglycan-β	SGCB-1
Sarcoglycan-δ,-γ	SGN-1
