# Supplementary material for: Wnt-Ror-Dvl signalling and the dystrophin complex organize planar-polarized membrane compartments in C. elegans muscles
Source: Nat Commun. 2024 Jun 10;15:4935. doi: 10.1038/s41467-024-49154-8 (PMC11164867; doi:10.1038/s41467-024-49154-8)
Supplement: Supplementary file 3 — Description of Additional Supplementary Files [file 41467_2024_49154_MOESM3_ESM.pdf]

<u>Description of Additional Supplementary Files</u>

File Name: Supplementary Data 1 List of C. elegans strains used in this study and corresponding figure panels.

File Name: Supplementary Data 2 List of C. elegans alleles used in this study.

File Name: Supplementary Data 3 List of single-strand oligonucleotides used in this study.

File Name: Supplementary Data 4 List of crRNA used for C. elegans CRISPR/Cas9 gene editing in this study.

File Name: Supplementary Data 5 List of plasmids used in this study
